# The Circular RNA CircCOL1A1 Functions as a miR-149-5p Sponge to Regulate the Formation of Superior-Quality Brush Hair *via* the CMTM3/AR Axis

**DOI:** 10.3389/fcell.2022.760466

**Published:** 2022-02-02

**Authors:** Jian Wang, Xi Wu, Xiaomei Sun, Liuming Zhang, Qiang Wang, Jingwen Qu, Yanhu Wang, Yongjun Li

**Affiliations:** ^1^ College of Animal Science and Technology, Yangzhou University, Yangzhou, China; ^2^ Key Laboratory of Animal Genetics and Molecular Breeding of Jiangsu Province, Yangzhou University, Yangzhou, China

**Keywords:** circCOL1A1, miR-149-5p, CMTM3/AR axis, regulation, superior-quality brush hair formation

## Abstract

Superior-quality brush hair, also called Type III hair, can be obtained only from the cervical spine region of skin tissues of Yangtze River Delta white goats. The formation of superior-quality brush hair is controlled by a series of critical genes and related signaling pathways. Circular RNAs (circRNAs) are ubiquitous endogenous noncoding RNAs that regulate many biological and physiological processes in mammals. However, little is known about the potential regulatory role of circRNAs in superior-quality brush hair formation. Here, we analyzed circRNA sequencing data from cervical spine region skin tissues of normal-quality brush hair goats and superior-quality brush hair goats and then selected and identified the differentially expressed circRNA circCOL1A1. To investigate the regulatory role and mechanism of action of circCOL1A1, goat hair follicle stem cells (gHFSCs) were cultured and treated with a circCOL1A1 overexpression plasmid and small-interfering RNAs (siRNAs). Functional assays showed that circCOL1A1 knockdown promoted the proliferation and differentiation of gHFSCs cultured *in vitro* but inhibited stem cell apoptosis, whereas overexpression of circCOL1A1 suppressed stem cell proliferation and differentiation and induced apoptosis. Bioinformatics analysis combined with dual-luciferase reporter assays and RNA binding protein immunoprecipitation (RIP) verified that, mechanistically, circCOL1A1 could bind miR-149-5p directly and then relieve its inhibitory effect on CMTM3 to further control the CMTM3/AR axis. Collectively, our results reveal a novel regulatory pathway for the formation of superior-quality brush hair and indicate that circCOL1A1 plays a role in gHFSC growth and superior-quality brush hair formation by targeting the miR-149-5p/CMTM3/AR axis.

## Introduction

Yangtze River Delta white goats, also known as Haimen goats or Brush Hair goats, are an exceptional goat breed worldwide that can be used to harvest superior-quality brush hair (National livestock and Poultry Genetic Resources Committee, 2011). Superior-quality brush hair, also called Type-III hair, is famous for its pure whiteness, brilliant luster and fine elasticity characteristics (Guo et al., 2017; Wang et al., 2018). Previous studies have indicated that hair growth is closely associated with hair follicle development, and hair follicle stem cell proliferation, apoptosis and differentiation are critical for hair follicle development (Calvo-Sánchez et al., 2019; Xiao et al., 2020). Our existing research revealed that 1) androgen secretion and cold stress can stimulate the formation of superior-quality brush hair in Yangtze River Delta white goats, which can further promote and activate the expression of proteins (such as β-fibrinogen and keratin) and related signaling pathways (such as the Wnt/β-catenin pathway) known to participate in hair growth (Li et al., 2013; Yang et al., 2015). 2) Dual-specificity phosphatase 6 (DUSP6), S100 calcium-binding protein A (S100A), CKLF-like MARVEL transmembrane domain-containing 3 (CMTM3) and heat stress-associated genes (Hsp70) are important for regulating superior-quality brush hair traits (Ji et al., 2018). 3) A high methylation level of CMTM3 could promote the formation of superior-quality brush hair, and CMTM3, also known as CKLFSF3, could modulate the transcription level of androgen receptor (AR) (Zhong et al., 2006; Wang et al., 2018). 4) CMTM3 was confirmed as a target gene of miR-149-5p, and during superior-quality brush hair formation, miR-149-5p could regulate hair follicle stem cell proliferation and apoptosis by targeting the CMTM3/AR axis (Wang et al., 2020). 5) CMTM3 knockdown accelerates hair follicle stem cell proliferation and promotes AR expression, while overexpression of CMTM3 significantly inhibits the proliferation of stem cells and is accompanied by a reduction in the AR expression level (Wang et al., 2020; Wang et al., 2021). This critical evidence suggests that a series of protein-coding genes and noncoding RNAs (ncRNAs) are important for the formation of superior-quality brush hair.

Circular RNAs (circRNAs) are a major class of ncRNAs that have a covalently closed loop structure and lack 5′ caps and 3′ poly(A) tail sequences (Kulcheski et al., 2016; Meng et al., 2017). Because of their unique ring structure and the absence of free 5′ and 3’ ends, circRNAs are more stable and resistant to RNase R (ribonuclease R) than linear RNAs (Rong et al., 2017). Recent studies have revealed that circRNAs have several critical regulatory functions in eukaryotes, including acting as miRNA sponges (Wang C. et al., 2019), binding to proteins (Li et al., 2019), serving as templates for translation (Legnini et al., 2017), and regulating gene transcription (Li et al., 2015) or translation (Wu et al., 2019). Most circRNAs exert their function by acting as sponges of one or multiple miRNAs. For example, SRY and ciRS7 are known to function as competing endogenous RNAs (ceRNAs) to regulate gene transcription and posttranscriptional translation by binding to miR-138 and miR-7, respectively (Hansen et al., 2013; Jeck et al., 2013). In addition, an increasing number of studies have suggested that circRNAs are involved in tumorigenesis and tumor progression by regulating different biological processes. For example, circTLK1 plays an important regulatory role in the progression of renal cell carcinoma by binding to miR-136-5p and then promoting chromobox4 expression (Li et al., 2020). CircAGFG1 accelerates the proliferation, migration and invasion of breast cancer cells by sponging miR-195-5p (Yang et al., 2019). In livestock, circTTN and circMYBPC1 have been identified as ceRNAs that sponge miRNAs and modulate skeletal muscle development (Wang X. et al., 2019; Chen et al., 2021); circHIPK3 plays a critical role in the cow mammary gland to influence milk production by regulating the signal transducer and activator of transcription 5 (STAT5) signaling pathway (Liu et al., 2020). However, little is known about circRNA functions in Yangtze River Delta white goats and the regulatory mechanism involved in superior-quality brush hair formation.

In this study, we selected and characterized circRNA-circCOL1A1, which was obtained from our circRNA sequencing data for Yangtze River Delta white goat cervical spine skin tissues (unpublished). CircCOL1A1, derived from the back-splicing of collagen-I α1 (COL1A1) mRNA, is located on goat chromosome 19: 36112781-36113653 and is 873 nt long. We observed that circCOL1A1 expression was significantly downregulated and differentially expressed in cervical spine skin tissues between normal-quality (NQ) and superior-quality brush hair (SQ) goats. Furthermore, our results showed that circCOL1A1 suppresses hair follicle stem cell proliferation and differentiation and contributes to promoting stem cell apoptosis. Further regulatory mechanism assays revealed that circCOL1A1 functioned as a sponge of miR-149-5p and then promoted CMTM3 expression to affect the CMTM3/AR axis and control the formation of superior-quality brush hair. Overall, our studies may provide a novel regulatory mechanism involving circCOL1A1, miR-149-5p, CMTM3, and AR, making up a circCOL1A1/miR-149-5p/CMTM3/AR axis, in the formation of superior-quality brush hair, which may also promote the protection of Yangtze River Delta white goats as a germplasm resource.

## Materials and Methods

### Sample Collection and Ethics Statement

Yangtze River Delta white goats are also known as brush hair goats and Haimen goats. Heart, liver, spleen, lung, kidney, small intestine, longissimus dorsi, adipose, testicular, and skin tissues were collected from two different genealogies of Yangtze River Delta white goats, separately from three male NQ and three male SQ goats (aged 4,5 months, half sibling male goats in two different genealogies) at the Haimen State Goat Farm (Haimen City, Jiangsu Province, China). These tissues were immediately frozen in liquid nitrogen after harvesting. The materials and experimental procedures used in the study were approved by the Animal Care and Use Committee of Yangzhou University.

### CircRNA-miRNA Interaction Network Construction and Analysis

miRanda (3.3a) (http://www.miranda.org/), RNAhybrid (v2.1.2) (https://bibiserv.cebitec.uni-bielefeld.de/rnahybrid/), and Cytoscape (https://js.cytoscape.org/) software were employed to construct and visualize the circRNA-miRNA interaction network. The interaction network was based on our circRNA sequencing data (unpublished), which were obtained from cervical spine skin tissues in three NQ and three SQ goats. For the data handling, differentially expressed circRNAs between cervical spine skin tissues of NQ and SQ goats were identified and selected based on a criteria fold change (FC) ≥ 1.5 and false discovery rate (FDR) < 0.05. DESeq2 (https://www.bioconductor.org/), Gene Ontology (GO) (http://www.geneontology.org/), and KEGG (https://www.kegg.jp/) analyses were utilized to explore and identify the function and ceRNA mechanism of differentially expressed circRNAs in cervical spine skin tissues in the formation of superior-quality brush hair.

### CircRNA Selection and Specificity Identification

The circCOL1A1/miR-149-5p/CMTM3/AR axis was selected from the abovementioned constructed interaction network. RegRNA 2.0 (http://regrna2.mbc.nctu.edu.tw/index.html) and RNAhybrid (v2.1.2) software were used to obtain the secondary structure and information on the binding sites between circCOL1A1 and miR-149-5p. The miR-149-5p/CMTM3/AR axis was constructed and identified in our previous study (Wang et al., 2020). Sanger sequencing, PCR with divergent and convergent primers, RNase R treatment, actinomycin D treatment, and cytoplasmic and nuclear RNA purification assays were used to identify the structure and specificity of circCOL1A1. Two particular pairs of divergent and convergent primers (Supplementary Table S1) were designed to amplify circCOL1A1 and linear COL1A1 mRNA using cDNA and gDNA from cervical spine skin tissues as templates. CircAOL1A1 was only detected by specific divergent primers in the cDNA template, and then the product of divergent primer PCR was identified by Sanger sequencing analysis. Three micrograms of total RNA from cervical spine skin tissues was treated with 3 U/µg RNase R (Geneseed, Guangzhou, China) for 10 min at 37°C. Then, hair follicle stem cells from male Yangtze River Delta white goats were cocultured with growth medium and 2 μg/ml actinomycin D (Sigma-Aldrich, Saint Louis, United States). In addition, cytoplasmic and nuclear RNA from hair follicle stem cells was independently extracted using a cytoplasmic and nuclear RNA purification kit (Norgen-Biotek, Thorold, Canada). These different RNA samples were isolated and transcribed into cDNA following the manufacturer’s instructions, followed by further evaluation of the structure and stability of circCOL1A1 and linear COL1A1 by the RT-qPCR assay. The OD260/280 values of these RNA samples were in the range from 1.80 to 2.00, and the RNA integrity number (RIN) values were in the range from 8.0 to 8.6, all of which met the experimental standards.

### Plasmid Construction and RNAi

Full-length circCOL1A1 (873 bp) was amplified and cloned into the pLC5-ciR circRNA overexpression vector (Geneseed, Guangzhou, China) to overexpress circCOL1A1. siRNAs to specifically target and interfere with the junction sites of circCOL1A1 were designed and synthesized by Geneseed (Guangzhou, China). The wild-type and mutant binding sites of circCOL1A1 with miR-149-5p were cloned into the psiCHECK-2™ vector (Promega, Madison, United States) to construct circCOL1A1-wild-type (psiCHECK-2-wild-circCOL1A1) and circCOL1A1-mut (psiCHECK-2-mutant-circCOL1A1) vectors using the *Xho*I and *Not*I restriction sites, respectively. A miR-149-5p sensor with two repeated complementary sequences that completely matched the mature sequence of miR-149-5p was created and inserted into the psiCHECK-2™ vector downstream of the hRluc luciferase locus. All constructed vectors were further confirmed by Sanger sequencing (Sangon, Shanghai, China). Primer sequence information for plasmid construction and RNAi is listed in Supplementary Table S1.

### Cell Culture and Transfection

Hair follicle stem cells from Yangtze River Delta white goats were isolated from newborn male goats’ neck skin, and the collection and culture procedures were described in our previous study (Wang Q. et al., 2019; Wang et al., 2020). Hair follicle stem cells were cultured in 6-well plates (Corning, New York, United States) with 2 ml growth medium (GM) consisting of DMEM-F12 (Gibco, New York, United States) supplemented with 20% fetal bovine serum (FBS) (Gibco, New York, United States) and 2% penicillin-streptomycin (Invitrogen, CA, United States) in each well and incubated at 37°C in an atmosphere containing 5% CO_2_. HEK293T cells were cultured with 2 ml GM consisting of DMEM-F12 supplemented with 10% FBS and 1% penicillin-streptomycin at 37°C in an atmosphere containing 5% CO_2_.

The effect of circCOL1A1 on hair follicle stem cell proliferation and apoptosis was investigated by transfecting hair follicle stem cells with the circCOL1A1 overexpression vector and circCOL1A1 siRNA oligos using Lipofectamine 3,000 (Invitrogen, CA, United States) following the manufacturer’s instructions. The miR-149-5p mimics and inhibitors were synthesized by and purchased from Gene Pharma (Suzhou, China) (Supplementary Table S2). A synthetic β-catenin gene overexpression vector (Sangon Biotech, Shanghai, China) was employed to induce hair follicle stem cell differentiation. Transfection was performed when the cultured stem cells grew to ∼70-80% confluence. The circCOL1A1 overexpression vector and circCOL1A1 siRNA oligos were separately transfected into stem cells when induced hair follicle stem cells stably overexpressed β-catenin. After transfection, the cells were incubated and cultured in Opti-MEM (Gibco, New York, United States) for 6 h, after which the transfection medium was replaced with fresh GM for 48 h. Hair follicle stem cells were induced to differentiate for up to 1 day and 7 days. All hair follicle stem cell cultures were performed at least in triplicate.

### Cell and Tissue RNA Isolation, Reverse Transcription PCR (RT-PCR), and Real-Time Quantitative PCR (RT-q-PCR)

Total RNA was extracted from hair follicle stem cells and collected tissues using a TRIzol kit (Takara, Tokyo, Japan). For gene and circCOL1A1 quantification, total RNA was reverse-transcribed into cDNA using the Takara Prime-Script RT kit with gDNA eraser (Tokyo, Japan). For miR-149-5p quantification, stem-loop and oligo-primers (dT-17) were used for reverse-transcription. RT-q-PCR was performed in triplicate on an ABI 7500/7500-Fast Real-Time PCR system (Applied Biosystems, CA, United States) with Takara TB Green II Master Mix Reagent Kit (Tokyo, Japan). GAPDH (for gene and circCOL1A1 quantification) and 18S-rRNA and U6-snRNA (for miR-149-5p quantification) were selected as the internal normalization controls. The relative gene, circCOL1A1 and miR-149-5p expression abundances were calculated using the 2^−ΔΔCt^ method (Arocho et al., 2006; Adnan et al., 2011). Primer sequences for RT-PCR and RT-q-PCR are listed in Supplementary Table S3.

### Western Blotting

Total cellular protein was extracted from each treatment group using RIPA lysis buffer (Solarbio, Beijing, China) supplemented with 1% PMSF (Solarbio, Beijing, China) and then quantified using a BCA protein assay kit (Solarbio, Beijing, China). Twenty micrograms of protein was separated via 8% or 10% SDS-polyacrylamide gel electrophoresis gels and subsequently transferred to polyvinylidene fluoride (PVDF) membranes (Immobilon, Darmstadt, Germany). The membranes were then blocked with 5% skim milk (Sangon Biotech, Shanghai, China) and incubated overnight at 4°C with primary antibodies (including antibodies against PCNA, CDK1, CCND2, Bcl2, Bax, Caspase 3, Caspase 9, CMTM3, β-catenin, C-myc, KRT6 and β-actin). Primary antibodies included the following targets: PCNA (MW: 29 kDa, Abcam, Cambridge, United Kingdom, CN: ab18197, 1:1000 dilution), CDK1 (MW: 34 kDa, Abcam, Cambridge, United Kingdom, CN: ab32384, 1:1000 dilution), CCND2 (MW: 33 kDa, Abcam, Cambridge, United Kingdom, CN: ab207604, 1:1000 dilution), Bcl2 (MW: 26 kDa, Proteintech, Rosemont, IL, United States, CN: 12789-1-AP, 1:1000 dilution), Bax (MW: 21 kDa, Abcam, Cambridge, United Kingdom, CN: ab32503, 1:1000 dilution), Caspase3 (MW: 32 kDa, Proteintech, Rosemont, IL, United States, CN:19677-1-AP, 1:1000 dilution), Caspase9 (MW: 46 kDa, Proteintech, Rosemont, IL, United States, CN: 10380-1-AP, 1:1000 dilution), CMTM3 (MW: 20 kDa, Bioss, Beijing, China, CN: bs-8021R, 1:1000 dilution), β-catenin (MW: 86 kDa, Abcam, Cambridge, United Kingdom, CN: ab32572, 1: 5000 dilution), C-myc (MW: 49 kDa, Abcam, Cambridge, United Kingdom, CN: ab32072, 1: 1000 dilution), KRT6 (MW: 60 kDa, Abcam, Cambridge, United Kingdom, CN: ab93279, 1: 2000 dilution) and β-actin (MW: 42 kDa, Abcam, Cambridge, United Kingdom, CN:ab8229, 1:500 dilution). Then, the membranes were washed and incubated with horseradish peroxidase-conjugated secondary antibodies (including goat-specific anti-rabbit IgG, CN: BS13278 and rabbit-specific anti-goat IgG, Bioworld, Nanjing, China, CN:BS30503, 1:5000 dilution). Finally, the protein bands were visualized with Super-Enhanced ECL Reagent (Biosharp, Hefei, China) and then analyzed on a Bio-Rad ChemiDocTM Touch Imaging System (Bio-Rad, CA, United States). Next, the band intensities on the images were further analyzed using Image Lab (v5.2.1) software (Bio-Rad, CA, United States).

### Cell Proliferation, Cell Cycle, and Cell Apoptosis Assays

For the cell proliferation assay, hair follicle stem cells were seeded at a density of 1 × 10^5^ cells/well in 24-well plates (Corning, New York, United States) with GM and transfected. After transfection, the stem cells were incubated for 48 h with serum-free medium containing EdU reagent from an EdU cell proliferation kit (RiboBio, Guangzhou, China). Then, fluorescent images were collected using a Leica fluorescence microscope (Leica, Wetzlar, Germany), and the imaging parameters were identical in all fluorescence microscopy images. For cell cycle and apoptosis assays, hair follicle stem cells were separately seeded at a density of 5 × 10^5^ cells/well in 6-well plates for 48 h after transfection. The treated stem cells for the cell cycle assay were harvested and incubated with propidium iodide (PI) staining buffer (Beyotime, Shanghai, China) and then subjected to flow cytometry analysis (FACSAria SORP, BD BioSciences, NJ, United States). For the cell apoptosis assay, the treated stem cells were double-conjugated with Annexin V-FITC staining buffer (Beyotime, Shanghai, China) and PI staining buffer, and then the cells were analyzed using flow cytometry. ModFit LTTM software (Verity Software House, Topsham, ME, United States) and CytExpert v2.3 software (Beckman Coulter, CA, United States) were individually applied to analyze and obtain cell cycle and cell apoptosis histograms.

### Dual-Luciferase Assay

HEK293T cells were seeded and cultured at a density of 1 × 10^5^ cells/well in 24-well plates with GM for transfection. The circCOL1A1-wild-type (containing the circCOL1A1 wild-type sequence) reporter plasmid and circCOL1A1-mut (containing the circCOL1A1 mutant-type sequence) plasmid were further confirmed by Sanger sequencing. Then, the circCOL1A1 wild-type or mutant reporter plasmid and miR-149-5p oligos (mimics, inhibitors and biosensor) were co-transfected into HEK293T cells. Forty-eight hours after transfection, the cells were lysed, and then Firefly and Renilla luciferase activities were examined on a BioTek Synergy 2 Multimode Microplate Reader (BioTek, VT, United States) using a Dual-Luciferase Reporter Assay kit (TransGen, Beijing, China).

### RNA Binding Protein Immunoprecipitation (RIP) Assay

The RIP assay was performed with the RNA-Binding Protein Immunoprecipitation Kit (Geneseed, Guangzhou, China) following the manufacturer’s instructions. A total of 1 × 10^7^ hair follicle stem cells were seeded and grown to ∼70–80% confluence. Then, the stem cells were collected and lysed using RIP lysis buffer. Subsequently, the pooled lysates were equally divided into two parts for incubation overnight at 4°C with immunoglobulin G (IgG) antibody (Millipore, Massachusetts, United States) and anti-Argonaute-2 (Ago-2) antibody (Abcam, Cambridge, United Kingdom). Then, the immunoprecipitated RNA was extracted and purified. Finally, the expression abundance of circCOL1A1 and miR-149-5p in the pull-down fractions was evaluated by the RT-q-PCR assay.

### Immunofluorescence and Immunohistochemistry Assay

Hair follicle stem cells were induced to differentiate for 7 days with different treatments and then rinsed with phosphate buffered saline (PBS, Solarbio, Beijing, China) three times. Subsequently, the stem cells were fixed with 4% paraformaldehyde for 30 min at room temperature and further permeabilized for 15 min with PBS supplemented with 0.5% Triton X-100 reagent. Stem cells were stained overnight at 4°C using anti-β-catenin, anti-C-myc, and anti-KRT6 immunofluorescence staining antibodies (Abcam, Cambridge, United Kingdom). The next day, we incubated these cells with goat anti-rabbit IgG H&L (Alexa Fluor ^®^ 488 or ^®^ 647) secondary antibodies (Abcam, Cambridge, United Kingdom) for 1 h at room temperature. Stem cell nuclei were stained with Hoechst-33342 staining solution (Beyotime, Shanghai, China). Finally, the stained cells were rinsed with PBS three times away from light, and then the fluorescence images were observed and collected using a Leica fluorescence microscope (Leica, Wetzlar, Germany). For the immunohistochemistry assay, paraffin sections of cervical spine skin tissues were incubated with primary antibodies, including anti-β-catenin, C-myc and KRT6 antibodies, overnight at 4°C. Afterward, a biotin-labeled secondary antibody (Boster, Wuhan, China) was used for further incubation for 30 min at 37°C. Finally, the paraffin sections were stained using 3, 3′-diaminobenzidine tetrahydrochloride (DAB) (Boster, Wuhan, China) and then collected and observed using an Olympus CX31/CX33 microscope (Olympus, Tokyo, Japan).

### Statistical Analysis

All data produced in this study are expressed as the mean ± standard error of the mean (SEM) and are based on at least three or six independent biological replicates for each assay. One-way ANOVA was performed with SPSS v24 software (IBM, Armonk, NY, United States) to analyze circCOL1A1 and linear COL1A1 expression levels in the collected tissues. Independent-samples T-tests were performed with SPSS v24 and Origin 7.5 software (OriginLab, MA, United States) to analyze and compare different treatment groups. *p*-values < 0.05 were significant, and *p*-values < 0.01 were exceedingly significant. ^*^ *p* < 0.05 and ^**^ *p* < 0.01.

## Results

### Construction and Analysis of Differentially Expressed circRNA-miRNA Networks

**FIGURE 1:**
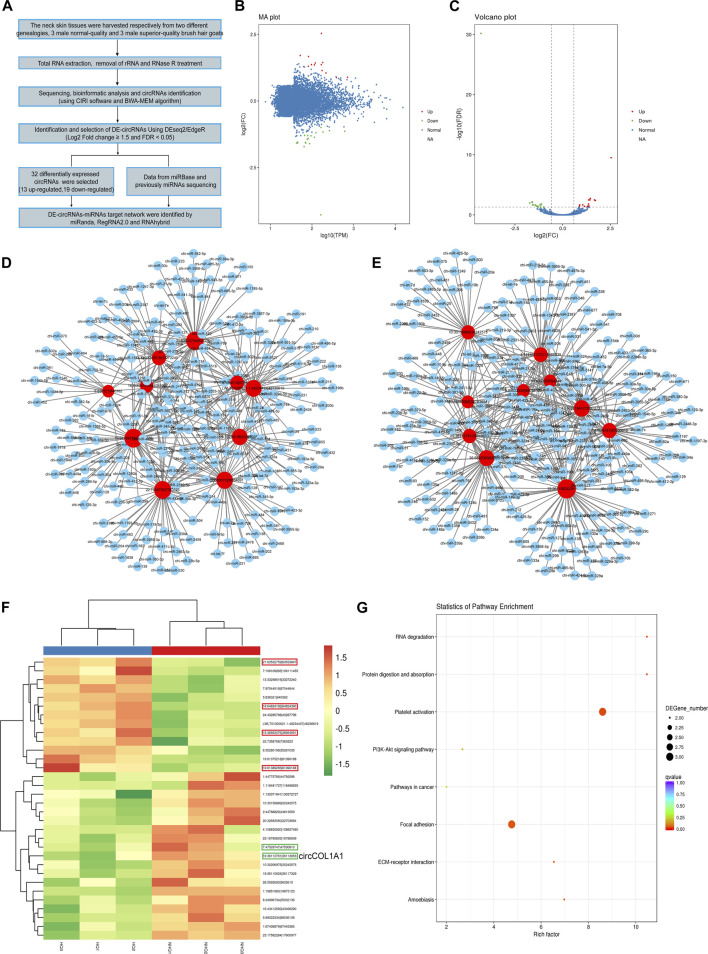
Differential expression circRNA-miRNA network construction and analysis. **(A)** Thumbnail of the data analysis. **(B,C)** MA plot **(B)** and volcano plot **(C)** of 32 differentially expressed circRNAs between NQ and SQ goats selected based on a log2-fold change ≥1.5 and FDR < 0.05. **(D)** Diagram of the top 10 upregulated circRNA-miRNA interaction networks. **(E)** Diagram of the top 10 downregulated circRNA-miRNA interaction networks. **(F)** Heatmap of 32 differentially expressed circRNAs identified by comparing NQ and SQ goats. **(G)** KEGG analysis of the host genes of differentially expressed circRNAs.

After simple collection, preprocessing, sequencing, and data analysis, 32 differentially expressed circRNAs were selected (13 upregulated and 19 downregulated). The information is listed below. The expression level of the circRNAs was normalized and calculated using transcripts per million (TPM) (TPM: Read_Count×1,000,000 ÷ Mapped_Reads). To identify the differentially expressed circRNAs in the skin tissues between NQ and SQ goats, the differentially expressed circRNAs (DE-circRNAs) were screened and obtained with the DESeq2 package. CircRNAs with a FC ≥ 1.5 and FDR < 0.05 were deemed to be differentially expressed (Figure 1A). The 13 upregulated circRNAs are shown in red in the MA and volcano plots, and the 19 downregulated circRNAs are shown in green (Figures 1B,C). In the data analysis, the top 10 upregulated circRNA-miRNA interaction networks (Figure 1D) and the top 10 downregulated circRNA-miRNA interaction networks (Figure 1E) were constructed. In these networks, the red nodes represent up or downregulated circRNAs, and the blue nodes represent the interacting miRNAs. Moreover, the deeper red color and larger node size indicate higher expression of the circRNAs. The 13 upregulated and 19 downregulated circRNAs are also displayed in the heatmap (Figure 1F). CircCOL1A1, the fourth most downregulated circRNA, originates from the COL1A1 gene, which encodes the pro-α1 chains of collagen-I (referenced by NCBI and GeneCards). Kyoto Encyclopedia of Genes and Genomes (KEGG) pathway analysis also showed that the host genes of the differentially expressed circRNAs were mostly enriched in the focal adhesion and platelet activation pathways (Figure 1G). These results suggested that the identified interaction networks might be closely related to superior-quality brush hair formation.

### CircCOL1A1 Identification, Characterization, and Expression Profiling

**FIGURE 2:**
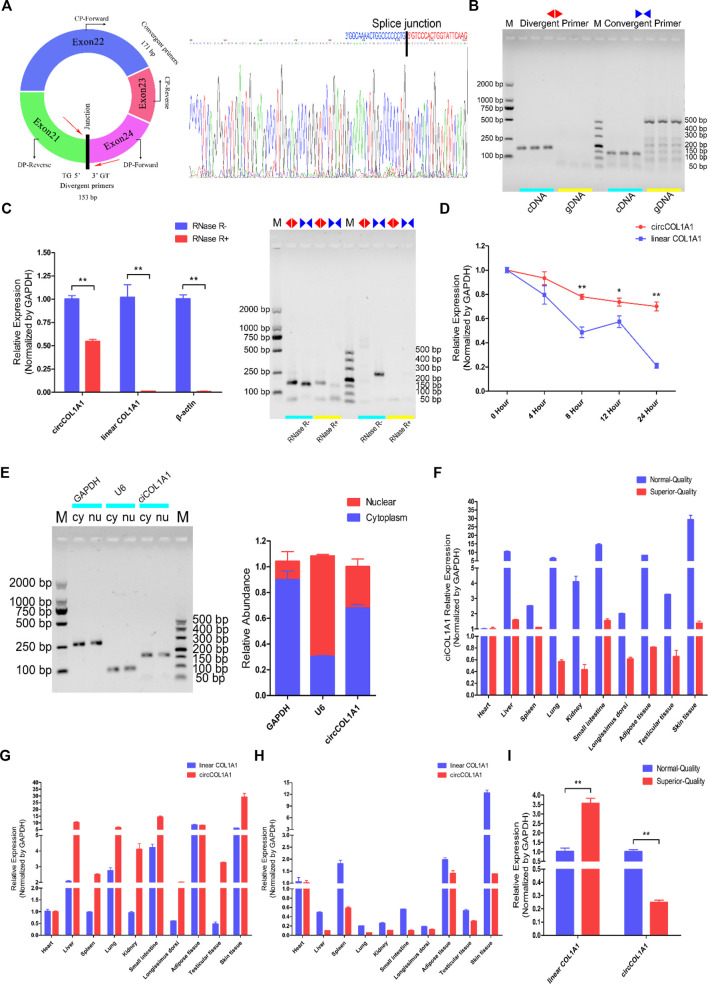
Identification, characterization, and expression profile of circCOL1A1. **(A)** The structure and splice junction sites of goat circCOL1A1 were identified by Sanger sequencing. **(B)** PCR with divergent and vonvergent primers confirmed the existence of circCOL1A1. **(C,D)** RNase R **(C)** and actinomycin D treatment **(D)** combined with RT-qPCR assays indicated that the abundance and stability of circCOL1A1 was greater than that of linear COL1A1 mRNA. **(E)** The expression of circCOL1A1 was detected in stem cell nuclei and cytoplasm. **(F)** Expression levels of circCOL1A1 in 10 different tissues in NQ and SQ goats. **(G)** Expression levels of circCOL1A1 and linear COL1A1 mRNA in 10 different tissues in NQ goats. **(H)** Expression levels of circCOL1A1 and linear COL1A1 mRNA in 10 different tissues in SQ goats. **(I)** Expression abundance of circCOL1A1 and linear COL1A1 mRNA in cervical spine skin tissues between NQ and SQ goats.

CircCOL1A1 originates from the COL1A1 gene, which is located on chromosome 19 in the goat genome, and exons 21, 22, 23, and 24 generate a head-to-tail closed cyclic structure (Figure 2A). Then, the splice junction sites of circCOL1A1 were confirmed by Sanger sequencing (Figure 2A). To identify the structure of circCOL1A1, we designed specific divergent primers for circCOL1A1 and convergent primers for linear COL1A1. The PCR products of cDNA and genomic DNA (gDNA) were resolved by 1% agarose gel electrophoresis, and the divergent primers successfully amplified the predicted band from the cDNA template but not from the gDNA template, while the band amplified by the convergent primers was obtained with both the cDNA and gDNA templates (Figure 2B). In addition, the RNase R treatment results combined with RT-qPCR and 1% agarose gel electrophoresis indicated that circCOL1A1 was resistant to RNase R, with an observed reduction in circCOL1A1 expression by approximately half, and the expression of linear COL1A1 or β-actin was not observed (Figure 2C). Moreover, the actinomycin-D treatment assay further showed that the half-life of circCOL1A1 was longer and the structure was more stable than that of linear COL1A1 (Figure 2D). To investigate the localization of circCOL1A1 in hair follicle stem cells, cytoplasmic RNA and nuclear RNA were independently extracted. Further RT-qPCR assays revealed that circCOL1A1 was mainly located in the cytoplasm of stem cells (Figure 2E), suggesting that circCOL1A1 might perform its function via the “ceRNA” mechanism. We also found that circCOL1A1 expression was extensive and differential in various normal-quality goat and superior-quality goat tissues (Figure 2F). The expression of circCOL1A1 was higher than that of linear COL1A1 in various tissues in normal-quality goats (Figure 2G). Conversely, linear COL1A1 expression was higher than circCOL1A1 expression in superior-quality goats (Figure 2H). Furthermore, consistent with our circRNA sequencing data, the expression of circCOL1A1 was significantly lower in superior-quality goat skin tissues than in normal-quality goat tissues (Figure 2I). Together, these results indicated that circCOL1A1 was a real circRNA and might regulate superior-quality brush hair formation.

### CircCOL1A1 Negatively Regulates Goat Hair Follicle Stem Cell Proliferation

**FIGURE 3:**
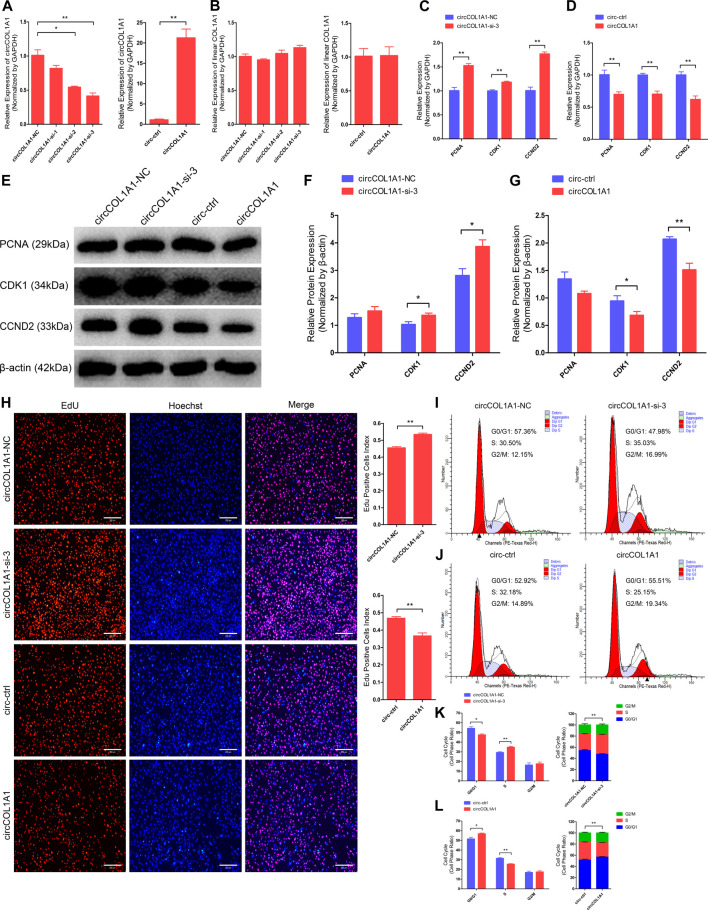
CircCOL1A1 negatively regulates goat hair follicle stem cell proliferation. **(A)** CircCOL1A1 was successfully suppressed or overexpressed in hair follicle stem cells after transfection with the circCOL1A1-siRNAs and overexpression plasmid for 48 h. **(B)** Linear COL1A1 mRNA was not suppressed or overexpressed in hair follicle stem cells after transfection with the circCOL1A1-siRNAs and overexpression plasmid for 48 h **(C,D)** Relative mRNA expression of PCNA, CDK1, and CCND2 at 48 h after transfection with circCOL1A1-si-3 **(C)** and circCOL1A1-overexpressing plasmid **(D)**. **(E,F,G)** Protein expression of PCNA, CDK1, and CCND2 at 48 h after transfection with circCOL1A1-si-3 and circCOL1A1-overexpressing plasmids. **(H)** Representative images of the EdU assay at 48 h after transfection. Bars, 200 µm. **(I,J,K,L)** The cell cycle distribution was detected at 48 h after transfection by flow cytometry **(I,J)**, and the distribution was determined **(K,L)**.

To investigate the regulatory role of circCOL1A1 in the formation of superior-quality brush hair, we first determined the role of circCOL1A1 during hair follicle stem cell proliferation. CircCOL1A1 expression in hair follicle stem cells was significantly inhibited or increased by transfection with junction site-specific circCOL1A1-siRNAs (siRNA-2 and siRNA-3) and circCOL1A1 overexpression vector (circCOL1A1) for 48 h (Figure 3A). Linear COL1A1 expression was not reduced or increased (Figure 3B), so siRNA-3 (circCOL1A1-si-3) and circCOL1A1 (overexpression vector) were selected for subsequent assays. First, stem cell RNA and protein were collected for RT-qPCR and western blotting to assess the expression of proliferation indicators (PCNA, CDK1 and CCND2). CircCOL1A1-si-3 enhanced the mRNA (Figure 3C) and protein (Figures 3E,F) levels of proliferation indicators. Conversely, circCOL1A1 reduced the mRNA (Figure 3D) and protein (Figures 3E,G) levels of proliferation indicators. Second, a 5-ethynyl-2′-deoxyuridine (EdU) cell proliferation assay of stem cells revealed that circCOL1A1-si-3 significantly increased the proportion of EdU-positive stem cells, while circCOL1A1 overexpression notably decreased this proportion (Figure 3H). Moreover, the cell cycle assay by flow cytometry further suggested that circCOL1A1-si-3 promoted stem cell cycle arrest in G0/G1-phase and then increased the stem cell number at S-phase (Figures 3I,K), while circCOL1A1 enhanced the proportion of stem cells in G0/G1-phase and decreased the cell number in S-phase (Figures 3J,L). These results indicated that circCOL1A1 suppressed goat hair follicle stem cell proliferation.

### CircCOL1A1 Facilitates Goat Hair Follicle Stem Cell Apoptosis

**FIGURE 4:**
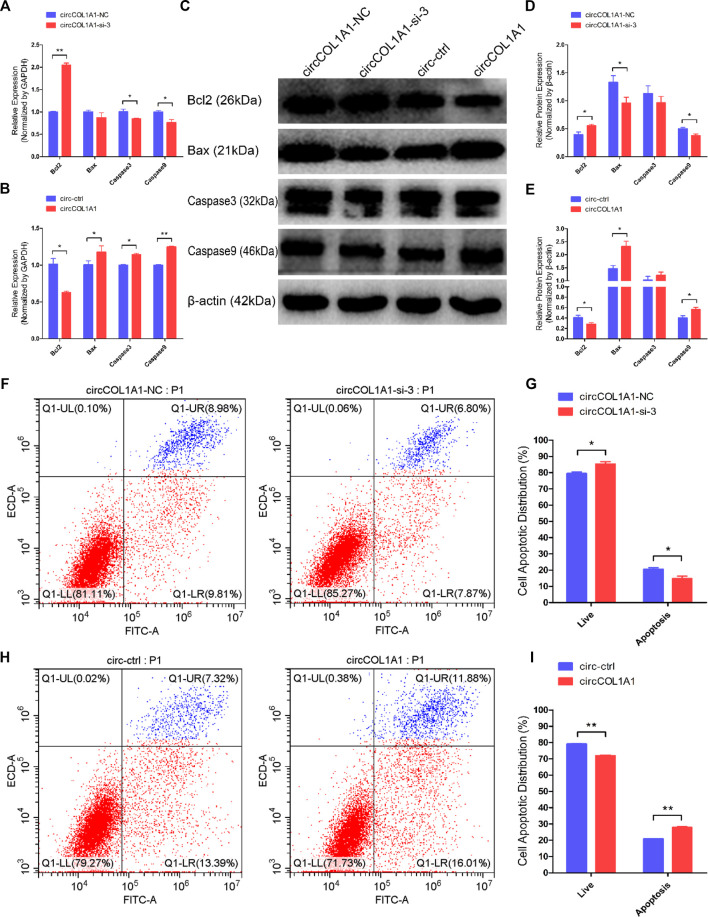
CircCOL1A1 facilitates goat hair follicle stem cell apoptosis. **(A,B)** The relative mRNA expression of Bcl2, Bax, Caspase3, and Caspase9 at 48 h after transfection with circCOL1A1-si-3 **(A)** and circCOL1A1-overexpressing plasmid **(B)**. **(C)** Protein expression of Bcl2, Bax, Caspase3, and Caspase9 at 48 h after transfection with circCOL1A1-si-3 and circCOL1A1-overexpressing plasmid. **(D,E)** Relative protein expression of Bcl2, Bax, Caspase3, and Caspase9 at 48 h after transfection. **(F,G)** The cell apoptosis rate was detected at 48 h after transfection with circCOL1A1-si-3 by Annexin V-FITC/PI double staining followed by the flow cytometry assay. **(H,I)** The cell apoptosis rate was detected at 48 h after transfection with the circCOL1A1-overexpressing plasmid by Annexin V-FITC/PI double staining followed by the flow cytometry assay.

Next, we explored the role of circCOL1A1 in stem cell apoptosis. RT-qPCR and western blotting assays were applied to detect the expression of antiapoptotic gene (Bcl2) and proapoptotic genes (Bax, Caspase3 and Caspase9). As shown in Figures 4A–E, circCOL1A1-si-3 markedly promoted the expression of Bcl2 and inhibited the expression of Bax, Caspase3, and Caspase9 (Figures 4A,C,D). In contrast to knockdown of circCOL1A1, circCOL1A1 overexpression strongly suppressed Bcl2 expression and enhanced Bax, Caspase3, and Caspase9 expression (Figures 4B,C,E). However, the mRNA level of Bax after circCOL1A1-si-3 treatment and the protein level of caspase3 after both circCOL1A1 inhibition and overexpression did not show significant changes. In addition, Annexin V-FITC/propidium iodide (PI) apoptosis staining assays further revealed that circCOL1A1 had a proapoptotic influence on stem cells. As shown in Figures 4F–I, circCOL1A1-si-3 protected stem cells from apoptosis and diminished the proportion of apoptotic cells (Figures 4F,G), while circCOL1A1 induced apoptosis and increased the proportion of apoptotic cells (Figures 4H,I). Together, these results demonstrated that circCOL1A1 facilitated goat hair follicle stem cell apoptosis.

### CircCOL1A1 Suppresses β-Catenin-Induced Goat Hair Follicle Stem Cell Differentiation

To determine the role of circCOL1A1 in hair follicle stem cell differentiation, we successfully constructed a β-catenin-overexpressing plasmid and harvested hair follicle stem cells overexpressing β-catenin. The expression levels of stem cell differentiation markers (β-catenin, C-myc and KRT6) were analyzed by RT-qPCR and immunofluorescence assays. As shown in Supplementary Figure S1, the RNA levels of β-catenin, C-myc, and KRT6 were obviously increased in β-catenin-induced hair follicle stem cells after 1 day (Supplementary Figure S1A) and 7 days (Supplementary Figure S1B) of culture. Immunofluorescence assays also showed that the β-catenin-overexpressing plasmid could induce goat hair follicle stem cell differentiation (Supplementary Figure S2). Therefore, we used hair follicle stem cells overexpressing β-catenin in the subsequent study.

**FIGURE 5:**
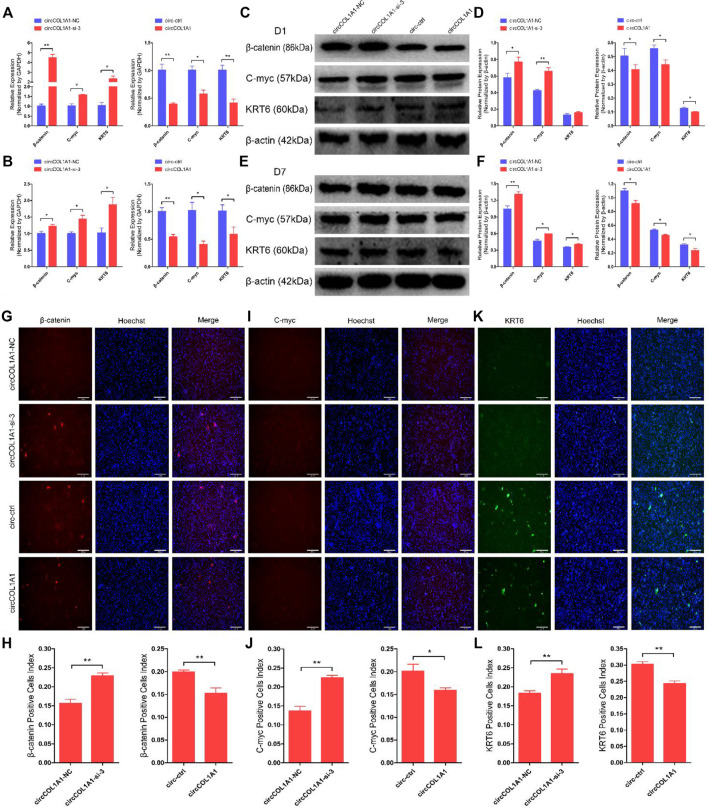
CircCOL1A1 suppresses β-Catenin-induced goat hair follicle stem cell differentiation. **(A,B)** The relative mRNA expression of β-catenin, C-myc, and KRT6 at 1 day **(A)** and 7 days **(B)** after transfection with circCOL1A1-si-3 and circCOL1A1-overexpressing plasmid in β-catenin-induced hair follicle stem cells. **(C,D)** Protein expression of β-catenin, C-myc, and KRT6 at 1day after transfection with circCOL1A1-si-3 and circCOL1A1-overexpressing plasmid in β-catenin-induced hair follicle stem cells. **(E, F)** Protein expression of β-catenin, C-myc, and KRT6 at 7 days after transfection with circCOL1A1-si-3 and circCOL1A1-overexpressing plasmid in β-catenin-induced hair follicle stem cells. **(G,H)** β-Catenin protein expression during β-catenin-induced hair follicle stem cell differentiation was detected after 7 days using an immunofluorescence assay after transfection with circCOL1A1-si-3 and circCOL1A1-overexpressing plasmid. **(I,J)** C-myc protein expression during β-catenin-induced hair follicle stem cell differentiation was detected after 7 days using an immunofluorescence assay after transfection with circCOL1A1-si-3 and circCOL1A1-overexpressing plasmid. **(K,L)** KRT6 protein expression during β-catenin-induced hair follicle stem cell differentiation was detected after 7 days using an immunofluorescence assay after transfection with circCOL1A1-si-3 and circCOL1A1-overexpressing plasmid.

To ascertain the functional role of circCOL1A1 in β-catenin-induced hair follicle stem cell differentiation, we transfected circCOL1A1-si-3 and the circCOL1A1 overexpression vector (circCOL1A1) into β-catenin-induced hair follicle stem cells and then cultured them for 1 day and 7 days. RT-qPCR (Figure 5A) and western blotting (Figures 5C,D) assays showed that circCOL1A1-si-3 promoted the expression of β-catenin, C-myc, and KRT6, while circCOL1A1 repressed the expression of these marker genes after culturing for 1 day. The expression levels of β-catenin, C-myc and KRT6 were also increased or decreased by transfection of circCOL1A1-si-3 or circCOL1A1, respectively, followed by culturing for up to 7 days (Figures 5B,E,F). To further visualize the expression of β-catenin, C-myc, and KRT6 in β-catenin-induced hair follicle stem cells, we also performed an immunofluorescence assay. The data revealed that circCOL1A1-si-3 accelerated the expression of β-catenin (Figures 5G,H), C-myc (Figure 5J), and KRT6 (Figures 5K,L), and this effect was reversed by circCOL1A1 overexpression. Together, these results indicated that circCOL1A1 could suppress β-catenin-induced goat hair follicle stem cell differentiation. In addition, immunohistochemistry-paraffin (IHC-P) assays showed that the expression of stem cell differentiation markers was higher in the cervical spine region of skin tissues in superior-quality brush hair than in normal-quality brush hair (Supplementary Figure S3A and Supplementary Figure S3B), which further suggested that the higher expression levels of β-catenin, C-myc, and KRT6 could be beneficial to the formation of superior-quality brush hair.

### CircCOL1A1 Acts as a Sponge for miR-149-5p in Hair Follicle Stem Cells

**FIGURE 6:**
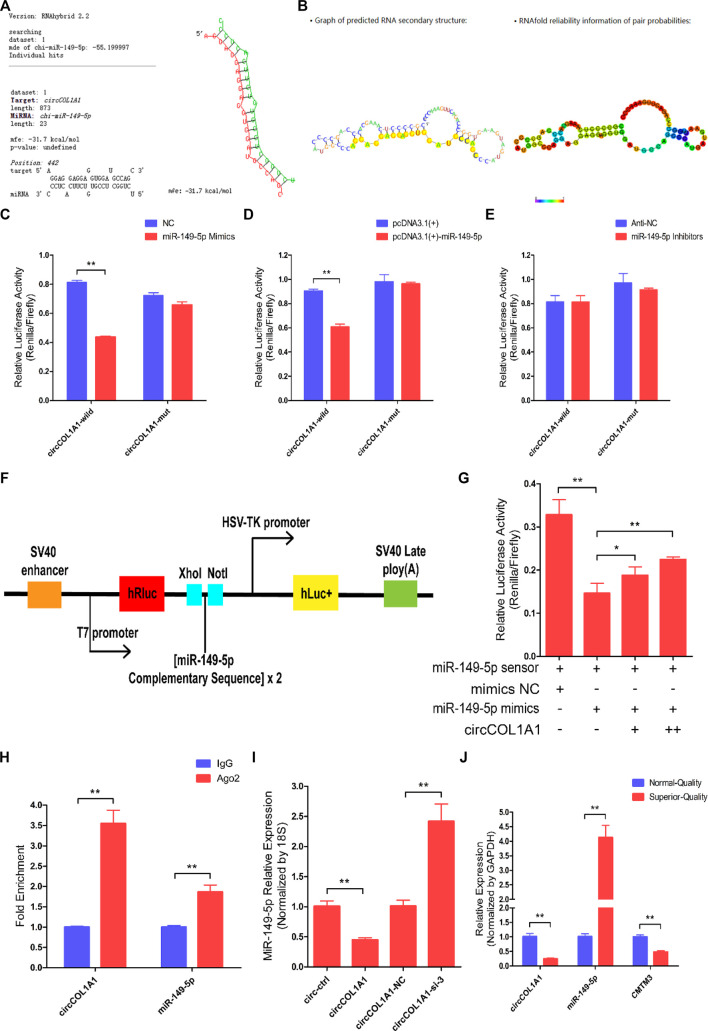
CircCOL1A1 acts as a sponge for miR-149-5p in goat hair follicle stem cells. **(A)** The predicted circCOL1A1 binding site sequence on miR-149-5p using RNAhybrid (v2.1.2) software. **(B)** The predicted secondary structure and base pair probabilities for circCOL1A1 and miR-149-5p using RegRNA v2.0 software. **(C)** miR-149-5p mimics were co-transfected with the circCOL1A1-wild-type or circCOL1A1-mut vector into HEK293T cells. **(D)** The pcDNA3.1 (+)-miR-149-5p plasmid was co-transfected with the circCOL1A1-wild-type or circCOL1A1-mut vector into HEK293T cells. **(E)** The miR-149-5p Inhibitors were co-transfected with the circCOL1A1-wild-type or circCOL1A1-mut vector into HEK293T cells. **(F)** A schematic diagram of the miR-149-5p biosensor structure. **(G)** The miR-149-5p biosensor was co-transfected with miR-149-5p mimics or circCOL1A1-overexpressing plasmid into 293T cells. **(H)** Fold enrichment of circCOL1A1 and miR-149-5p determined by RT-q-PCR following the RIP assay with Ago-2 and IgG antibodies (negative control). **(I)** The expression of miR-149-5p after circCOL1A1 overexpression and inhibition using the RT-q-PCR assay. **(J)** Expression abundance of circCOL1A1, miR-149-5p, and CMTM3 in cervical spine skin tissues from NQ and SQ goats.

To explain the potential regulatory mechanism of circCOL1A1 during the formation of superior-quality brush hair, the circCOL1A1/miR-149-5p axis was identified from our sequencing data and the predicted interactional networks. Therefore, we speculated that circCOL1A1 might function as a ceRNA to regulate miRNA expression (Hansen et al., 2013; Memczak et al., 2013), and the shared binding sites between circCOL1A1 and miR-149-5p were obtained using RNAhybrid-v2.1.2 software (Figure 6A). The secondary structure and base-pairing information were visualized using RegRNA-v2.0 software (Figure 6B). Dual-luciferase assays showed that miR-149-5p overexpression treatment (by cotransfection of mimics and the pcDNA3.1 (+)-miR-149-5p plasmid with the circCOL1A1-wild-type plasmid) notably inhibited luciferase activity, while cotransfection with the circCOL1A1-mut plasmid did not change the luciferase activity (Figures 6C,D). miR-149-5p inhibitor treatment (by cotransfection of inhibitors with the circCOL1A1-wild-type or circCOL1A1-mut plasmid) also did not alter the luciferase activity (Figure 6E). To further determine whether circCOL1A1 functions as a sponge to sequester miR-149-5p, a miR-149-5p sensor was generated by copying two repeated miR-149-5p complementary sequences into the psiCHECK-2™ vector (Figure 6F). The results showed that the luciferase activity was markedly reduced in the miR-149-5p mimics and miR-149-5p sensor cotransfection groups, while this reduction could be partly restored by adding different doses of the circCOL1A1 overexpression vector (Figure 6G). The sponge mechanism between circCOL1A1 and miR-149-5p was further evaluated and confirmed using RNA immunoprecipitation (RIP) and RT-qPCR assays. The results showed that circCOL1A1 and miR-149-5p were successfully enriched in the argonaute-2 (Ago-2) protein pull-down group but not in the immunoglobulin G (IgG) protein pull-down group (Figure 6H). In addition, the expression levels of miR-149-5p in hair follicle stem cells were decreased or increased after transfection with circCOL1A1 (overexpression plasmid) and circCOL1A1-si-3, respectively (Figure 6I). The relationship between miR-149-5p and its target gene CMTM3 was confirmed in our previous study (Wang et al., 2020). On this basis, we investigated the expression trends of circCOL1A1, miR-149-5p, and CMTM3 in cervical spine skin tissues of NQ and SQ goats. Interestingly, the expression of circCOL1A1 and CMTM3 showed the same trend, while miR-149-5p showed the opposite trend (Figure 6J), suggesting that circCOL1A1 sponged miR-149-5p to enhance CMTM3 expression. Altogether, these findings confirmed that circCOL1A1 functioned as a “sponge” to interact with miR-149-5p.

### CircCOL1A1 Suppresses Goat Hair Follicle Stem Cell Proliferation by Sponging miR-149-5p

**FIGURE 7:**
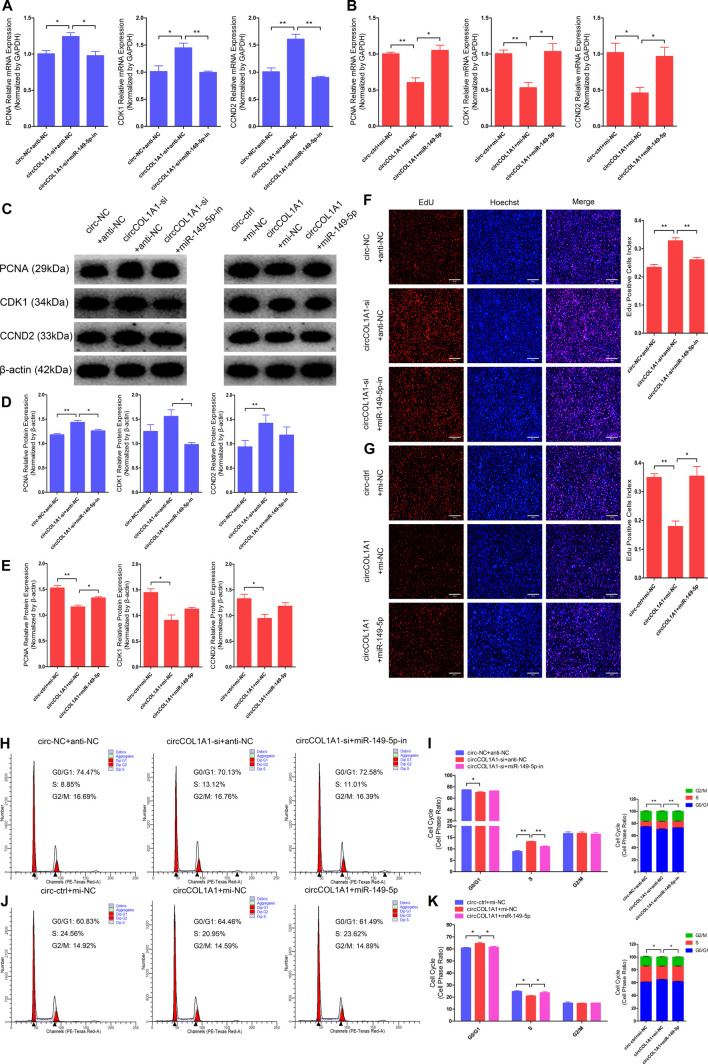
Effects of circCOL1A1 and miR-149-5p cotransfection on the proliferation of goat hair follicle stem cells. **(A)** The relative expression of proliferation markers (PCNA, CDK1, and CCND2) at 48 h after hair follicle stem cells were co-transfected with circCOL1A1-si (siRNA-3) and miR-149-5p-in (inhibitors). **(B)** The relative expression of proliferation markers after 48 h in stem cells co-transfected with circCOL1A1 (overexpressing plasmid) and miR-149-5p (mimics). **(C,D)** Protein expression of proliferation markers after 48 h in stem cells co-transfected with circCOL1A1-si and miR-149-5p-in. **(C,E)** Protein expression of proliferation markers after 48 h in stem cells co-transfected with circCOL1A1 and miR-149-5p. **(F,G)** Representative images of the EdU assay at 48 h after circCOL1A1-si and miR-149-5p-in cotransfection **(F)** and circCOL1A1 and miR-149-5p cotransfection **(G)**. Bars, 200 µm. **(H,I)** The cell cycle distribution was detected at 48 h after cotransfection with circCOL1A1-si and miR-149-5p-in by the flow cytometry assay. **(J, K)** The cell cycle distribution was detected at 48 h after cotransfection with circCOL1A1 and miR-149-5p by the flow cytometry assay.

Our previous study showed that miR-149-5p is important for the formation of superior-quality brush hair and could promote goat hair follicle stem cell proliferation and inhibit stem cell apoptosis by downregulating CMTM3 expression at the posttranscriptional level (Wang et al., 2020). Therefore, we hypothesized that circCOL1A1 might function as a sponge in goat hair follicle stem cells in a CMTM3-dependent manner by competitively binding to miR-149-5p. RT-qPCR and western blotting assays showed that circCOL1A1 knockdown (circCOL1A1-si + anti-NC) significantly increased the mRNA (Figure 7A) and protein (Figures 7C,D) levels of PCNA, CDK1, and CCND2, and this effect was relieved by cotransfection with miR-149-5p inhibitors (circCOL1A1-si + miR-149-5p-in). In contrast, as shown in Figures 7B,C,E, circCOL1A1 overexpression (circCOL1A1+mi-NC) obviously inhibited proliferation marker gene expression; however, this inhibition was rescued by cotransfection with miR-149-5p mimics (circCOL1A1+miR-149-5p) at both the transcriptional and translational levels. Second, the EdU and cell cycle assays indicated that circCOL1A1 knockdown clearly facilitated hair follicle stem cell proliferation, accompanied by a higher EdU-positive stem cell index (Figure 7F), increased the number of stem cells at S-phase, and decreased the proportion of cells in G0/G1-phase (Figures 7H,I), and the EdU-positive stem cell index and S-phase stem cell number were markedly reduced after cotransfection with miR-149-5p inhibitors. Conversely, circCOL1A1 overexpression clearly reduced the EdU-positive stem cell index (Figure 7G), increased the number of stem cells at G0/G1-phase, and decreased the proportion of cells in S-phase (Figures 7J,K), and the reduction could be reversed after cotransfection with miR-149-5p mimics. Together, these results indicated that the inhibition of hair follicle stem cell proliferation by circCOL1A1 could be rescued by using miR-149-5p.

### CircCOL1A1 Facilitates Goat Hair Follicle Stem Cell Apoptosis by Sponging miR-149-5p

**FIGURE 8:**
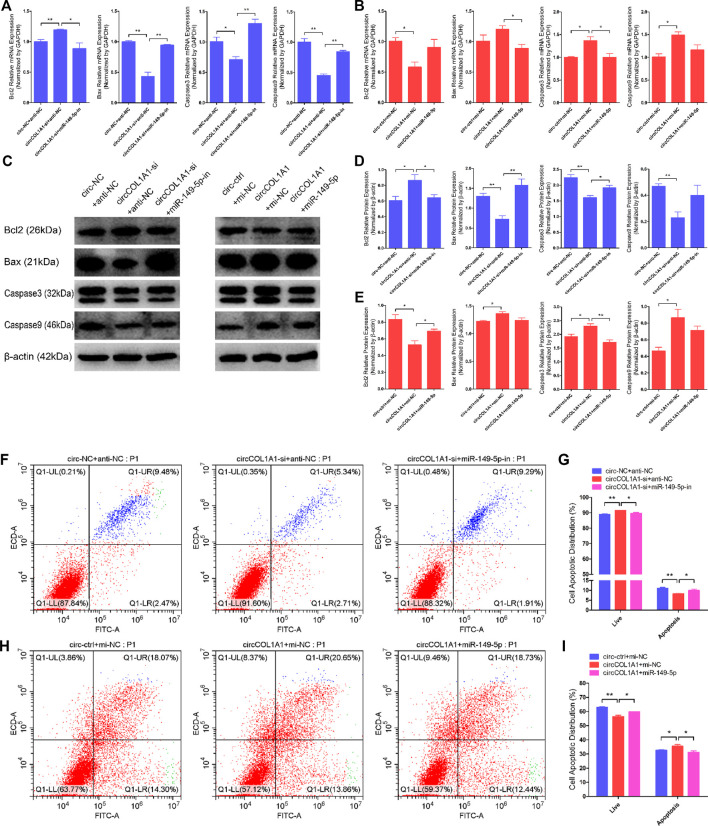
CircCOL1A1 and miR-149-5p cotransfection influenced goat hair follicle stem cell apoptosis. **(A)** The relative expression of antiapoptotic gene (Bcl2) and proapoptotic genes (Bax, Caspase3 and Caspase9) in hair follicle stem cells co-transfected with circCOL1A1-si and miR-149-5p-in for 48 h. **(B)** The relative expression of antiapoptotic gene and proapoptotic genes in hair follicle stem cells co-transfected with circCOL1A1 and miR-149-5p for 48 h **(C,D)** Protein expression of antiapoptotic gene and proapoptotic genes in hair follicle stem cells co-transfected with circCOL1A1-si and miR-149-5p-in for 48 h **(C,E)** Protein expression of antiapoptotic gene and proapoptotic genes in hair follicle stem cells co-transfected with circCOL1A1 and miR-149-5p for 48 h **(F,G)** The cell apoptosis rate was detected at 48 h after cotransfection with circCOL1A1-si and miR-149-5p-in by Annexin V-FITC/PI double staining followed by the flow cytometry assay. **(H,I)** The cell apoptosis rate was detected at 48 h after cotransfection with circCOL1A1 and miR-149-5p by Annexin V-FITC/PI double staining followed by the flow cytometry assay.

Next, we explored the role of circCOL1A1 and miR-149-5p cotransfection in stem cell apoptosis. RT-qPCR and western blotting assays showed that circCOL1A1 knockdown markedly promoted the expression of Bcl2 but inhibited the expression of Bax, Caspase3, and Caspase9 (Figures 8A,C,D), and these effects were reversed by cotransfection with the miR-149-5p inhibitors. In contrast to knockdown of circCOL1A1, circCOL1A1 overexpression markedly suppressed Bcl2 expression and facilitated Bax, Caspase3, and Caspase9 expression (Figures 8B,C,E), and apoptotic indicators expression was inhibited after cotransfection with miR-149-5p mimics. In addition, Annexin V-FITC/PI apoptosis staining assays revealed that circCOL1A1 knockdown inhibited stem cell apoptosis, accompanied by a decrease in the ratio of apoptotic cells, while stem cell apoptosis was promoted after cotransfection with miR-149-5p inhibitors (Figures 8F,G). In contrast, circCOL1A1 overexpression induced apoptosis and increased the proportion of apoptotic cells (Figures 4H,I), and this promotion of apoptosis was abolished after cotransfection with miR-149-5p mimics (Figures 8H,I). Together, these results demonstrated that circCOL1A1 facilitated hair follicle stem cell apoptosis and that this effect was relieved using miR-149-5p.

### CircCOL1A1 Promotes the Expression of CMTM3 by Sponging miR-149-5p

**FIGURE 9:**
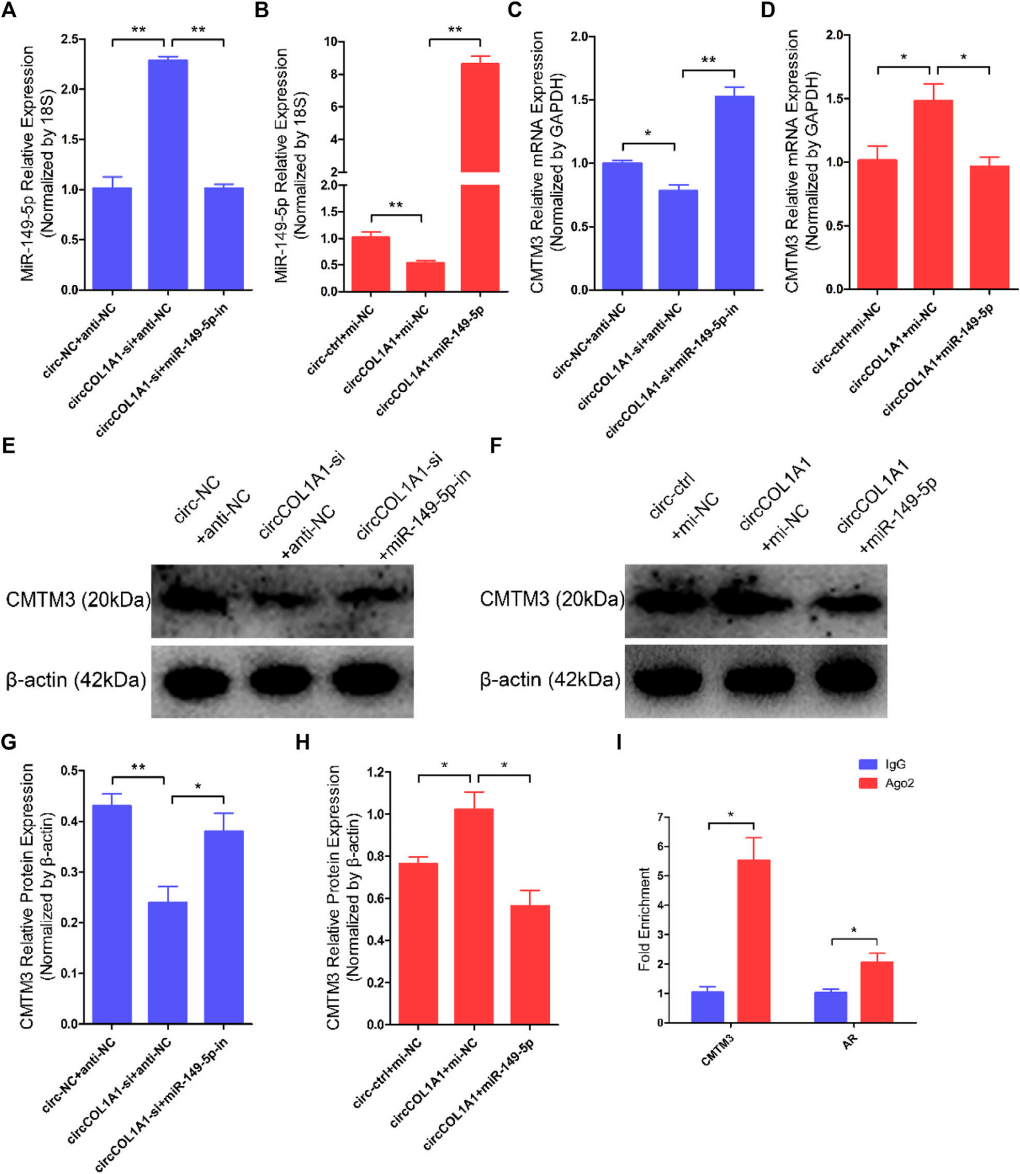
CircCOL1A1 regulates miR-149-5p and CMTM3 expression in goat hair follicle stem cells. **(A,B)** Expression of miR-149-5p in hair follicle stem cells after cotransfection with circCOL1A1-si and miR-149-5p-in **(A)** and with circCOL1A1 and miR-149-5p **(B)**. **(C,D)** The relative expression of CMTM3 in hair follicle stem cells after cotransfection with circCOL1A1-si and miR-149-5p-in (C) and with circCOL1A1 and miR-149-5p (D). **(E,G)** CMTM3 protein expression in hair follicle stem cells after cotransfection with circCOL1A1-si and miR-149-5p-in. **(F,H)** CMTM3 protein expression in hair follicle stem cells after cotransfection with circCOL1A1 and miR-149-5p. **(I)** Fold enrichment of CMTM3 and AR determined by RT-q-PCR following the RIP assay with Ago-2 antibody and IgG antibody (negative control).

**FIGURE 10:**
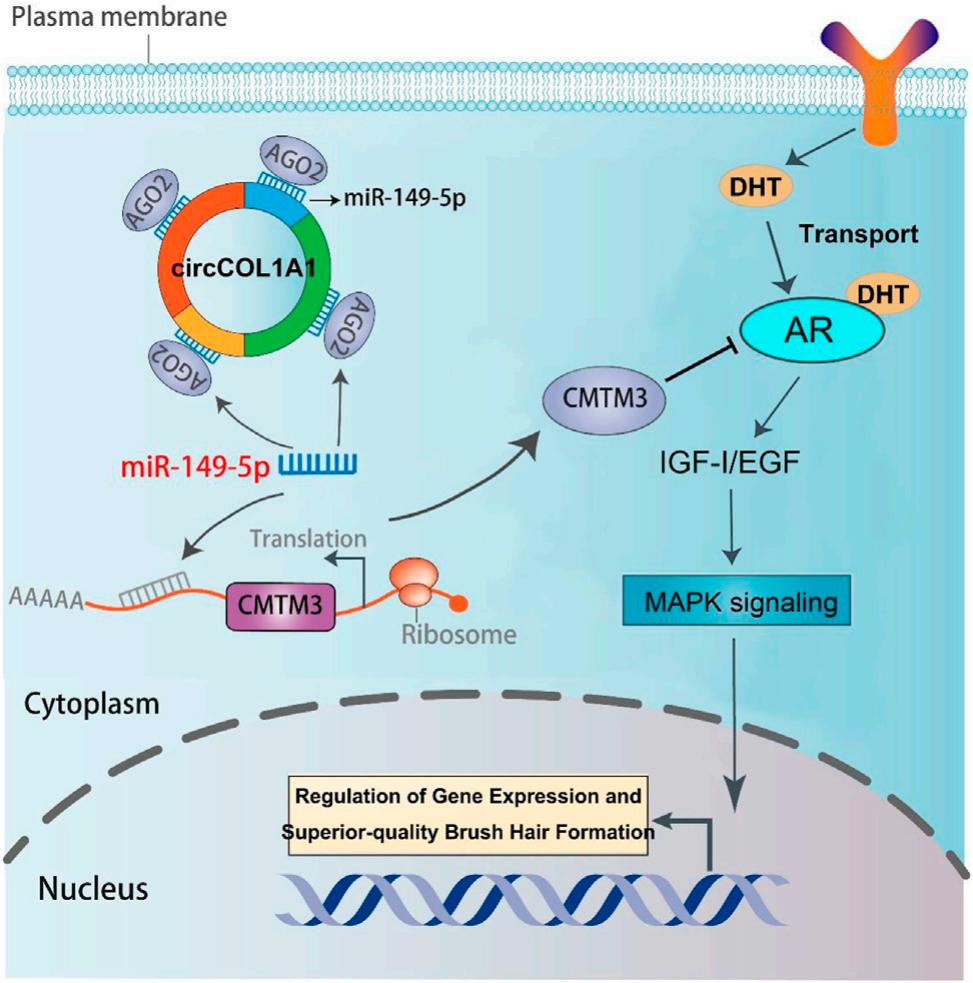
The regulatory model of the circCOL1A1/miR-149-5p/CMTM3/AR axis in goat hair follicle stem cells in superior-quality brush hair formation.

A regulatory mechanism involving miR-149-5p, CMTM3, and AR, in which miR-149-5p regulates goat hair follicle stem cell proliferation and apoptosis via the CMTM3/AR axis, further accelerates the formation of superior-quality brush hair (Wang et al., 2020). Based on our previous and current studies, we hypothesized that circCOL1A1 promotes CMTM3 expression by binding to miR-149-5p. RT-qPCR assay results showed that circCOL1A1 knockdown increased the expression levels of miR-149-5p, while circCOL1A1 overexpression inhibited miR-149-5p expression, and the expression of miR-149-5p could be downregulated or upregulated after cotransfection with miR-149-5p inhibitors or mimics, respectively (Figures 9A,B). CircCOL1A1 knockdown significantly inhibited the expression of CMTM3 at both the mRNA (Figure 9C) and protein (Figures 9E,G) levels, and the inhibition of CMTM3 expression was reversed after cotransfection with miR-149-5p inhibitors, which is contrary to the expression of miR-149-5p. Moreover, circCOL1A1 overexpression obviously promoted CMTM3 expression, and this promoting effect was reduced after cotransfection with miR-149-5p (Figures 9D,F,H), which is consistent with our previous studies. The relationship between CMTM3 and AR was further confirmed using RIP assays (Figure 9I). Finally, these results indicated that circCOL1A1 controls goat hair follicle stem cell proliferation, apoptosis and differentiation to further regulate the formation of superior-quality brush hair through the circCOL1A1/miR-149-5p/CMTM3/AR axis (Figure 10).

## Discussion

Recently, an increasing number of circRNAs have been confirmed to exist in human and domestic animal transcriptomes, and emerging research has indicated that circRNAs play important roles in regulating various cellular functions (Meng et al., 2017; Memczak et al., 2013), including cell proliferation, apoptosis, differentiation, migration, and invasion. In this study, differentially expressed circRNAs related to the superior-quality brush hair traits of Yangtze River Delta white goats were identified and annotated by circRNA sequencing and bioinformatics analysis. A novel circRNA, circCOL1A1, related to superior-quality brush hair formation was identified and characterized. In contrast to other ncRNAs (such as lncRNAs and miRNAs), circCOL1A1 has a more stable circular structure and a longer half-life period and is resistant to RNase R treatment, and its characteristics are in accordance with those of known, previously reported circRNAs (Patop et al., 2019). RT-qPCR and expression profile analyses showed that circCOL1A1 presented lower expression levels in the skin tissues of goats with superior-quality brush hair, but it had higher expression in the skin tissue of goats with normal-quality brush hair (Figures 2F–I). In addition, circCOL1A1 has been shown to be differentially expressed in various individuals and tissues, suggesting that circCOL1A1 may have an important role in functional regulation (Memczak et al., 2013). The location of circRNAs in cells can determine their regulatory functions. When located in the cell nucleus, circRNAs can act as molecular scaffolds to regulate gene transcription by recruiting key transcription factors and transcriptase (Li et al., 2015); when located in the cytoplasm, circRNAs can function as molecular sponges and compete with target genes for binding to certain miRNAs, which then relieves or releases miRNA-mediated inhibitory effects (degradation or translational suppression) on target gene mRNAs (Kristensen et al., 2018). In this study, we found that circCOL1A1 is mainly localized in the cytoplasm of stem cells by using nuclear and cytoplasmic RNA extraction and RT-qPCR assays, suggesting that circCOL1A1 could play its regulatory role by acting as a molecular sponge via a ceRNA mechanism. For example, circHUWE1 lightens its inhibitory effects on ATK3 by competitively combining bta-miR-29b and further controls myoblast development and cattle skeletal muscle myogenesis (Yue et al., 2020).

COL1A1 is a gene that encodes the pro-alpha-1 chains of collagen type-I, the triple helix of which comprises two alpha-1 chains and one alpha-2 chain. Collagen type-I is a valuable structural protein of the extracellular matrix that can be mostly found in connective tissues (Cole, 1994). In addition, COL1A1 has been identified as an oncogene that is involved in the carcinogenesis of colorectal cancer, gastric cancer (GC), and oral cancer and could be used as a potential therapeutic target (Li et al., 2016; He et al., 2018; Zhang et al., 2018). CircCOL1A1 is a novel circRNA and is derived from exons 21-24 of the COL1A1 gene. Our circRNA sequencing data indicate that circCOL1A1 is differentially expressed between NQ and SQ goats skin tissues. However, the role of circCOL1A1 has not been reported previously. In this study, we found that circCOL1A1 could suppress the proliferation and differentiation (β-catenin-induced stem cell differentiation) of goat hair follicle stem cells and facilitate stem cell apoptosis. The maintenance of stem cell numbers and the differentiation level of stem cells are essential for the formation of superior-quality brush hair, which is driven by many biological and physiological processes, especially stem cell proliferation and differentiation (Stenn and Paus, 2001; Wang et al., 2020). Proliferation assays showed that circCOL1A1 inhibition could facilitate stem cell proliferation by upregulating the expression of proliferation-related genes (PCNA, CDK1, and CCND2) and increasing the EdU-positive stem cell number and S-phase ratios of stem cells. In contrast, these promoting effects were inhibited by circCOL1A1 overexpression. Apoptotic assays indicated that circCOL1A1 overexpression promotes stem cell apoptosis by increasing the expression of apoptotic genes (Bax, Caspase3, and Caspase9), decreasing the expression of Bcl2 (antiapoptotic gene), and enhancing the rate of stem cell apoptosis. Conversely, these effects were reversed by circCOL1A1 inhibition. These phenomena showed that circCOL1A1 plays a negative role in the maintenance of stem cell numbers. Previous studies have shown that β-catenin can induce hair follicle stem cell differentiation by activating related downstream genes (such as C-myc and Bmp-4) and the Wnt/β-catenin signaling pathway (Nanba et al., 2013; Shen et al., 2017). In addition, the KRT6 gene has been shown to be involved in mediating goat hair follicle stem cell differentiation (Yan et al., 2019). Therefore, based on these reported studies and our differentiation assays (including RT-qPCR, western blotting, and stem cell immunofluorescence assays), we found that circCOL1A1 inhibition increases the expression levels of β-catenin, C-myc, and KRT6 in β-catenin-induced hair follicle stem cell differentiation, while circCOL1A1 overexpression decreases the expression of these stem cell differentiation factors (β-catenin, C-myc, and KRT6). Furthermore, immunohistochemistry assays of cervical spine skin tissues showed that β-catenin, C-myc, and KRT6 expression levels in superior-quality brush hair goat skin tissues are higher than those in NQ goat skin tissues. These assays suggest that circCOL1A1 also plays a negative role in hair follicle stem cell differentiation. Together, our results indicate that circCOL1A1 inhibits the formation of superior-quality brush hair.

CircRNAs, which have been identified as particular endogenous RNAs, have become novel members of the ceRNA family. This type of circRNA can act as a sponge to bind to and prevent miRNAs from binding to miRNA target genes (Qi et al., 2015). Here, the combined results of RT-qPCR, western blotting, RIP, and dual-luciferase reporter assays provided direct evidence that circCOL1A1 could function as a sponge for directly binding miR-149-5p to relieve the inhibitory effect of miR-149-5p on CMTM3 and further regulate the CMTM3/AR axis. Our previous studies have indicated that CMTM3 is a key gene that participates in the formation of superior-quality brush hair (Guo et al., 2017; Wang et al., 2018). Moreover, by targeting CMTM3, miR-149-5p accelerates the formation of superior-quality brush hair via upregulation of AR expression (Wang et al., 2020). In this study, we showed direct evidence that circCOL1A1 could reverse the inhibitory influence of miR-149-5p on CMTM3 expression and then control the CMTM3/AR axis (upregulation of CMTM3 expression and suppression of AR levels). Similar results have been reported for circNRIP1 in GC, which promotes GC cell proliferation, migration and invasion by acting as a miR-149-5p sponge and regulating the AKT1/mTOR axis (Zhang et al., 2019), and for circBICD2 in oral squamous cell carcinoma (OSCC), which functions as a sponge for miR-149-5p to modulate the miR-149-5p/IGF2BP1 axis in OSCC progression (Qiu et al., 2021). These results highlight that different circRNAs that function as sponges for miR-149-5p can exert various roles in molecular regulatory processes.

The miR-149-5p/CMTM3/AR axis was confirmed to play an important role during the formation of superior-quality brush hair in our previous study. Moreover, androgens have been verified to promote insulin-like growth factor-I (IGF-I) expression and act as regulators of hair follicle growth; androgens can also stimulate the formation of superior-quality brush hair and play their physiological role by binding to AR (Philpott et al., 1994; Wang et al., 2020). To our knowledge, this is the first study to construct a circRNA sponge regulatory network for superior-quality brush hair formation in Yangtze River Delta white goats. Thus, the exploration and identification of the circCOL1A1/miR-149-5p/CMTM3/AR axis in this study provides a promising target for examining whether Yangtze River Delta white goats can produce superior-quality brush hair.

## Conclusion

In conclusion, this study demonstrates the functions and regulatory mechanism of circCOL1A1 in hair follicle stem cells. Specifically, circCOL1A1 suppresses hair follicle stem cell proliferation and differentiation and induces stem cell apoptosis. Moreover, we revealed that circCOL1A1 plays roles in hair follicle stem cell growth and superior-quality brush hair formation by targeting the miR-149-5p/CMTM3/AR axis. Mechanistically, circCOL1A1 was confirmed to act as a sponge for miR-149-5p to then relieve the suppressive effect of miR-149-5p on its target CMTM3 and control the CMTM3/AR axis, which further inhibits the formation of superior-quality brush hair.

## Data Availability

The original contributions presented in the study are publicly available. This data can be found here: https://www.ncbi.nlm.nih.gov/bioproject/PRJNA778865/.

## Abbreviations

gHFSCs, goat hair follicle stem cells; siRNAs, small-interfering RNAs; IF, Immunofluorescence; IHC, immunohistochemistry; EdU, 5-ethynyl-2′-deoxyuridine; RIP, RNA binding protein immunoprecipitation; DUSP6, Dual-specificity phosphatase 6; S100A, S100 calcium-binding protein A; CMTM3, CKLF-like MARVEL transmembrane domain-containing 3; AR, androgen receptor; Hsp70, heat stress-associated genes; RNase R, ribonuclease R; ceRNAs, competing endogenous RNAs; STAT5, signal transducer and activator of transcription 5; COL1A1, collagen-I α1; GO, Gene Ontology; KEGG, Encyclopedia of Genes and Genomes; GM, growth medium; FBS, fetal bovine serum; RT-PCR, reverse transcription PCR; RT-q-PCR or RT-qPCR, real-time quantitative PCR; PVDF, polyvinylidene fluoride; PI, propidium iodide; IgG, immunoglobulin G; Ago2, Argonaute-2; DAB, 3, 3′- diaminobenzidine tetrahydrochloride; gDNA, genomic DNA; GC, gastric cancer; OCSC, oral squamous cell carcinoma; IGF-I, insulin-like growth factor-I; NQ, normal-quality brush hair; SQ, superior-quality brush hair; RIN, RNA integrity number.
