# Supplementary figures and images for: The Circular RNA CircCOL1A1 Functions as a miR-149-5p Sponge to Regulate the Formation of Superior-Quality Brush Hair via the CMTM3/AR Axis
Source: Front Cell Dev Biol. 2022 Feb 2;10:760466. doi: 10.3389/fcell.2022.760466 (PMC8847694; doi:10.3389/fcell.2022.760466)

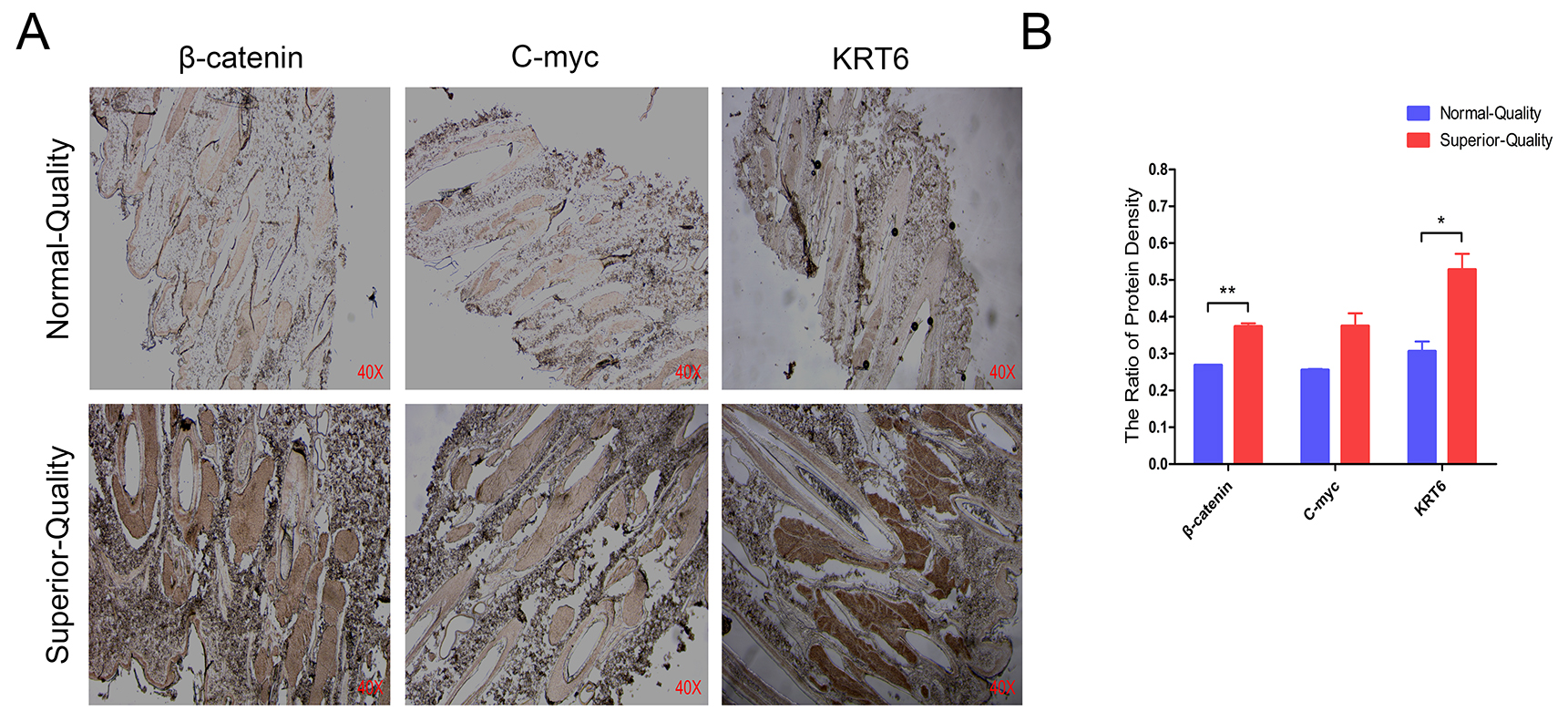

Supplement: Supplementary file 1 [file Image3.jpg]

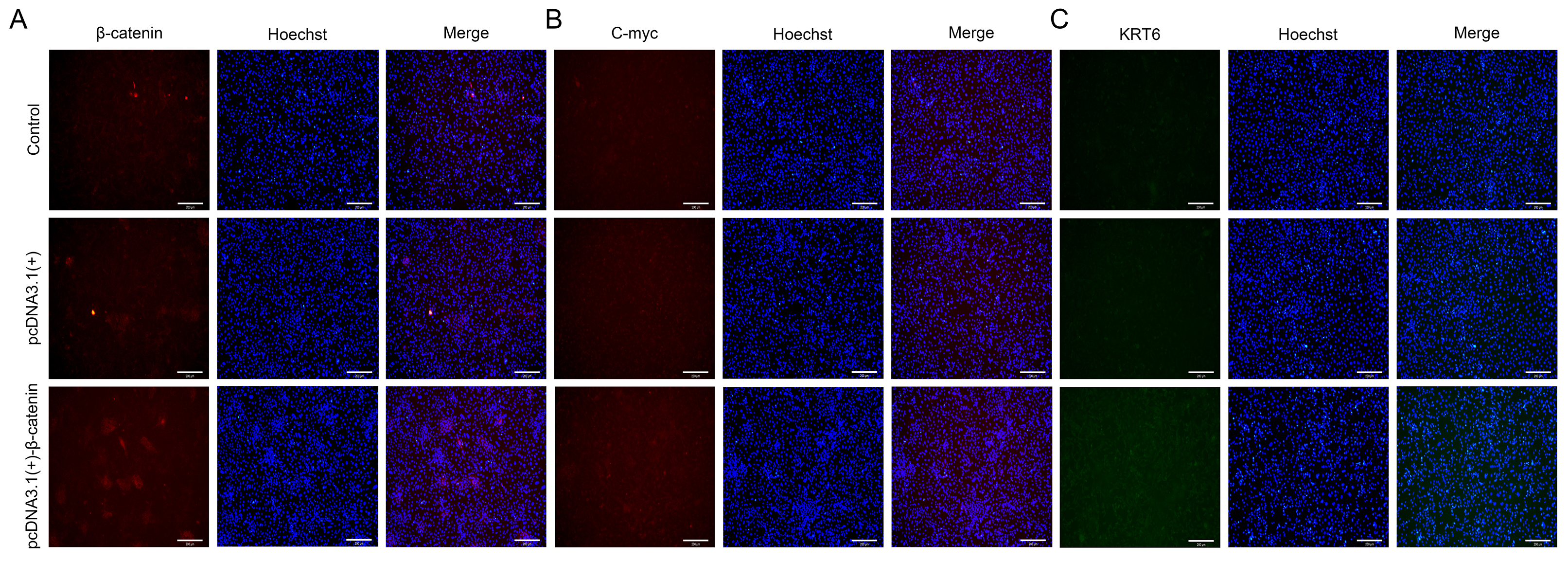

Supplement: Supplementary file 2 [file Image2.jpg]

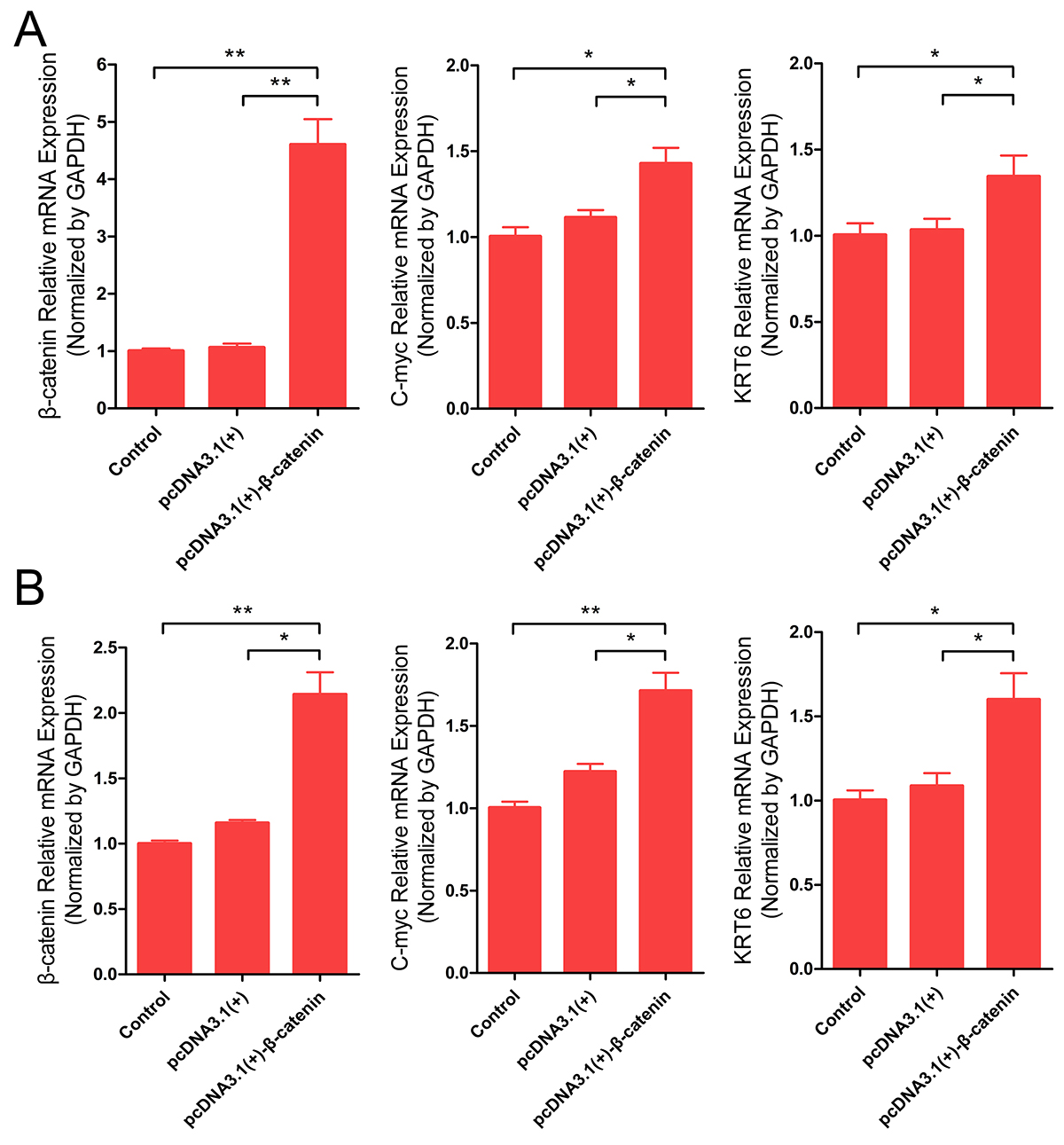

Supplement: Supplementary file 4 [file Image1.jpg]
