# Supplementary material for: The Circular RNA CircCOL1A1 Functions as a miR-149-5p Sponge to Regulate the Formation of Superior-Quality Brush Hair via the CMTM3/AR Axis
Source: Front Cell Dev Biol. 2022 Feb 2;10:760466. doi: 10.3389/fcell.2022.760466 (PMC8847694; doi:10.3389/fcell.2022.760466)
Supplement: Supplementary file 3 [file Table1.DOC]

Supplementary Information:

**TABLE S1**. Primer sequence information for circCOL1A1

| Name | Sequence Name | Sequence Information (5’ to 3’) |
| --- | --- | --- |
| Divergent primers | Divergent primer-F | TATCCTCCACTCCACTCACA |
|  | Divergent primer-R | CTTGAATACCAGTGGGACCAG |
| Convergent primers | Convergent primer-F | GGGGAAGAAGGAAAGCGAGG |
|  | Convergent primer-R | CTCACCAGCAGGACCCTTG |
| Si-NC and  siRNAs | CircCOL1A1-NC | TTCTCCGAACGTGTCACGTTT |
|  | CircCOL1A1-si-1 | AACTGGCCCCCCTGGTCCC |
|  | CircCOL1A1-si-2 | TGGCCCCCCTGGTCCCACT |
|  | CircCOL1A1-si-3 | CCCCCTGGTCCCACTGGTA |
| circCOL1A1-wild | circCOL1A1-wild-F | CCGCTCGAGTGACGGTGTTGCTGGCCCCAA |
|  | circCOL1A1-wild-R | ATAAGAATGCGGCCGCTCACCAGCAGGACCCTAGTT |
| circCOL1A1-mut | circCOL1A1-mut-F | CCGCTCGAGTGACGGTGTTGCTGGCCCCAA |
|  | circCOL1A1-mut-R | ATAAGAATGCGGCCGCTTCATCATGGCGACCGAAGGTGCAGGAGGAGGTGGGCCAT |
| miR-149-5p  sensor | miR-149-5p sensor-F | TCGAGGGGAGTGAAGACACGGAGCCAGAGGGAGTGAAGACACGGAGCCAGAGCGGCC |
|  | miR-149-5p sensor-R | GGCCGCTCTGGCTCCGTGTCTTCACTCCCTCTGGCTCCGTGTCTTCACTCCCCTCGA |

| **TABLE S2**. Sequence information for miR-149-5p oligos | | |
| --- | --- | --- |
| Name | Sequence Name | Sequence Information (5’ to 3’) |
|  | miR-149-5p Mimics | UCUGGCUCCGUGUCUUCACUCCC (sense) |
|  |  | GAGUGAAGACACGGAGCCAGAUU (antisense) |
|  | miR-149-5p NC | UUCUCCGAACGUGUCACGUTT (sense) |
| miR-149-5p |  | ACGUGACACGUUCGGAGAATT (antisense) |
|  | miR-149-5p Inhibitors | GGGAGUGAAGACACGGAGCCAGA |
|  | miR-149-5p Inhibitor NC | CAGUACUUUUGUGUAGUACAA |

**TABLE S3**. Primer sequence information for RT-PCR and RT-q-PCR

| Gene | Sequence Name | Sequence information (5’ to 3’) |
| --- | --- | --- |
| miR-149-5p | Stem-loop  RT-miR-149-5p^1^ | GTCGTATCCAGTGCAGGGTCCGAGGTATTCG  CACTGGATACGACGGGAGTGA |
|  | miR-149-5p Stem-loop-F  miR-149-5p Stem-loop-R | TCTGGCTCCGTGTCTTC |
|  |  | GTGCAGGGTCCGAGGT |
| 18S-rRNA  ID:493779 | 18S-rRNA-F  18S-rRNA-R | GTGGTGTTGAGGAAAGCAGACA |
|  |  | TGATCACACGTTCCACCTCATC |
| U6-snRNA  ID:19862 | U6-snRNA-F | GCTTCGGCACATATACTAAAAT |
|  | U6-snRNA-R | CGCTTCACGAATTTGCGTGTCAT |
| PCNA  ID:102172276 | PCNA-F | ATCAGCTCAAGTGGCGTGAA |
|  | PCNA-R | TGCCAAGGTGTCCGCATTAT |
| CDK1  ID:10086361 | CDK1-F | AGATTTTGGCCTTGCCAGAG |
|  | CDK1-R | AGCTGACCCCAGCAATACTT |
| CCND2  ID:102180657 | CCND2-F | GGGCAAGTTGAAATGGAA |
|  | CCND2-R | TCATCGACGGCGGGTAC |
| CMTM3  ID:102174055 | CMTM3-F | CCTCTGCTTCCTCTTTGCTGATG |
|  | CMTM3-R | ACGGCTGTGATGGAGATGGC |
| Bcl2  ID:100861254 | Bcl2-F | ATGTGTGTGGAGAGCGTCAA |
|  | Bcl2-R | CCTTCAGAGACAGCCAGGAG |
| BAX | BAX-F | TTTCCGACGGCAACTTCAA |
| ID:100846984 | BAX-R | TGAGCACTCCAGCCACAAA |
| Caspase3  ID:102177031 | Caspase3-F | AGGCAGACTTCTTGTACGCA |
|  | Caspase3-R | TTCTGTCGCTACCTTTCGGT |
| Caspase9  ID:102174681 | Caspase9-F | GGGGACTTCTGGTGGTTAGT |
|  | Caspase9-R | GAGTCAGGAGGGAGAAAGCTG |
| β-catenin  ID:102191742 | β-catenin-F | TGTTCGCCTTCACTACGGAC |
|  | β-catenin-R | TTGCTGGACAAAGGGCAAGA |
| C-myc  ID:102171262 | C-myc-F | ACGGAACTCTTGCGCCTAAA |
|  | C-myc-R | GCCAAGGTTGTGAGGTTGTTC |
| KRT6  ID:100860930 | KRT6-F | CAGTCCACTGTCTCTGGTGG |
|  | KRT6-R | CTGAAGCCACCTCCAATGCT |
| GAPDH  ID:100860872 | GAPDH-F | AGGTCGGAGTGAACGGATTC |
|  | GAPDH-R | CCAGCATCACCCCACTTGAT |

^1^ Stem-loop RT-miR-149-5p was applied for reverse transcription of miR-149-5p

**Supplementary Figure 1.** Expression of differentiation markers in β-Catenin-induced goat hair follicle stem cells. (**A**) The relative mRNA expression of β-catenin, C-myc and KRT6 at 1 day in β-catenin induced hair follicle stem cells. (**B**) The relative mRNA expression of β-catenin, C-myc and KRT6 at 7 days in β-catenin induced hair follicle stem cells.

**Supplementary Figure 2.** Fluorescent images of differentiation markers in β-Catenin-induced goat hair follicle stem cells. (**A**) Fluorescent images of β-catenin protein in β-catenin-induced hair follicle stem cells. (**B**) Fluorescent images of C-myc protein in β-catenin-induced hair follicle stem cells. (**C**) Fluorescent images of KRT6 protein in β-catenin-induced hair follicle stem cells.

**Supplementary Figure 3**. Expression of differentiation markers in skin tissues between normal-quality and superior-quality brush hair goats. (**A**) Higher β-catenin, C-myc and KRT6 expression in superior-quality brush hair goat skin tissues than in normal-quality brush hair goat skin tissues were observed by immunohistochemistry assay of-paraffin sections, magnification = 40X. (**B**) The density of β-catenin, C-myc and KRT6 proteins in the skin tissues between normal-quality brush hair goats and superior-quality brush hair goats.
